# The Technological Basis of a Balloon-Expandable TAVR System: Non-occlusive Deployment, Anchorage in the Absence of Calcification and Polymer Leaflets

**DOI:** 10.3389/fcvm.2022.791949

**Published:** 2022-03-03

**Authors:** Harish Appa, Kenneth Park, Deon Bezuidenhout, Braden van Breda, Bruce de Jongh, Jandré de Villiers, Reno Chacko, Jacques Scherman, Chima Ofoegbu, Justiaan Swanevelder, Michael Cousins, Paul Human, Robin Smith, Ferdinand Vogt, Bruno K. Podesser, Christoph Schmitz, Lenard Conradi, Hendrik Treede, Holger Schröfel, Theodor Fischlein, Martin Grabenwöger, Xinjin Luo, Heather Coombes, Simon Matskeplishvili, David F. Williams, Peter Zilla

**Affiliations:** ^1^Strait Access Technologies (SAT), University of Cape Town, Cape Town, South Africa; ^2^Cardiovascular Research Unit, University of Cape Town, Cape Town, South Africa; ^3^Chris Barnard Division for Cardiothoracic Surgery, University of Cape Town, Cape Town, South Africa; ^4^Department of Anaesthesia and Perioperative Medicine, University of Cape Town, Cape Town, South Africa; ^5^Deparment of Cardiac Surgery, Artemed Clinic Munich South, Munich, Germany; ^6^Department of Cardiac Surgery, Klinikum Nürnberg, Paracelsus Medical University, Nuremberg, Germany; ^7^Center for Biomedical Research, Medical University of Vienna, Vienna, Austria; ^8^Auto Tissue Berlin, Berlin, Germany; ^9^Department of Cardiac Surgery, University of Munich, Munich, Germany; ^10^Department of Cardiovascular Surgery, University Heart Center, Hamburg, Germany; ^11^Department of Cardiac and Vascular Surgery, University Hospital, Mainz, Germany; ^12^Department of Cardiovascular Surgery, University Heart Center, Freiburg, Germany; ^13^Department of Cardiovascular Surgery, Vienna North Hospital, Vienna, Austria; ^14^Department of Cardiac Sugery, Fu Wai Hospital, Peking Union Medical College, Beijing, China; ^15^Lomonosov Moscow State University Medical Center, Moscow, Russia; ^16^Wake Forest Institute of Regenerative Medicine, Wake Forest School of Medicine, Winston-Salem, NC, United States; ^17^Cape Heart Centre, University of Cape Town, Cape Town, South Africa

**Keywords:** balloon-expandable, plastic deformation, aortic regurgitations, polymer leaflets, rheumatic heart disease

## Abstract

Leaflet durability and costs restrict contemporary trans-catheter aortic valve replacement (TAVR) largely to elderly patients in affluent countries. TAVR that are easily deployable, avoid secondary procedures and are also suitable for younger patients and non-calcific aortic regurgitation (AR) would significantly expand their global reach. Recognizing the reduced need for post-implantation pacemakers in balloon-expandable (BE) TAVR and the recent advances with potentially superior leaflet materials, a trans-catheter BE-system was developed that allows tactile, non-occlusive deployment without rapid pacing, direct attachment of both bioprosthetic and polymer leaflets onto a shape-stabilized scallop and anchorage achieved by plastic deformation even in the absence of calcification. Three sizes were developed from nickel-cobalt-chromium MP35N alloy tubes: Small/23 mm, Medium/26 mm and Large/29 mm. Crimp-diameters of valves with both bioprosthetic (sandwich-crosslinked decellularized pericardium) and polymer leaflets (triblock polyurethane combining siloxane and carbonate segments) match those of modern clinically used BE TAVR. Balloon expansion favors the wing-structures of the stent thereby creating supra-annular anchors whose diameter exceeds the outer diameter at the waist level by a quarter. In the pulse duplicator, polymer and bioprosthetic TAVR showed equivalent fluid dynamics with excellent EOA, pressure gradients and regurgitation volumes. Post-deployment fatigue resistance surpassed ISO requirements. The radial force of the helical deployment balloon at different filling pressures resulted in a fully developed anchorage profile of the valves from two thirds of their maximum deployment diameter onwards. By combining a unique balloon-expandable TAVR system that also caters for non-calcific AR with polymer leaflets, a powerful, potentially disruptive technology for heart valve disease has been incorporated into a TAVR that addresses global needs. While fulfilling key prerequisites for expanding the scope of TAVR to the vast number of patients of low- to middle income countries living with rheumatic heart disease the system may eventually also bring hope to patients of high-income countries presently excluded from TAVR for being too young.

## Introduction

During several decades of development, transcatheter aortic valve replacement almost exclusively focused on the treatment of calcific aortic stenosis (AS) (1–4). Being the most common heart valve pathology in the Western World, it provided the patient numbers needed for non-inferiority studies in comparison with an established low-mortality procedure such as surgical aortic valve replacement (SAVR). Since in high-income countries (HIC) where TAVR was pioneered (1, 5), pure aortic regurgitation (AR) occurs less frequently than AS (4, 6, 7) pure AR did not have enough traction to influence developments. This is still reflected in contemporary TAVR designs whose simple mesh structures are sufficient to anchor the stents in the rigid calcific deposits of AS. However, given the huge global burden of rheumatic heart disease (RHD) in emerging economies (8–10) with its predominance of AR (9–13) and the growing number of patients with pure AR in industrialized countries, it seems timely to extend transcatheter procedures to patients with non-calcified regurgitant aortic valves (14–17).

Initial attempts to treat non-calcific pure AR with TAVR were directed at patients in HICs who were typically old, with reasonably preserved ventricular function (18). To compensate for the absence of calcification for anchoring, devices were distinctly oversized (19). The few newer generation devices with dedicated anchoring systems improved the success rates (18) but also highlighted how deployment requirements vary between AS and AR.

Further extending the indication for TAVR from patients with degenerative AR to those with RHD in low- to middle-income countries (LMICs) introduces additional challenges. These patients are significantly younger (20) and often present at a later stage of ventricular remodeling when they are past conventional operability. Severe volume overload, eccentric hypertrophy and excessive left ventricular (LV) wall stress cause progressive LV dysfunction, making it desirable to avoid rapid pacing during implantation (21). The hyperdynamic nature of eccentric hypertrophy also makes stabilization during deployment even more essential than in AS.

Thus, TAVRs that cater for this sizable but vulnerable group must address aspects that go beyond those of patients in industrialized countries that were hitherto also outside the spectrum of TAVR indications. The avoidance of rapid-pacing (21), of costly secondary procedures such as post-implant balloon dilatations (22) and of permanent pacemaker implantations (22, 23) are the foremost additional constraints in LMICs.

Guided by these considerations, we have developed a balloon-expandable (BE) TAVR system with several critical features. An hourglass shape was designed to ease the pressure on the conduction system. Expansion-linked plastic deformation of the stent was utilized to enable firm supra-annular anchorage in non-calcified roots. The stent has a continuous scallop design to allow the seamless attachment of degradation resistant polyurethane leaflets promising durability in younger patients through their fatigue- and calcification resistance. To avoid rapid pacing a helical hollow balloon was developed for the deployment system protecting against backflow through a temporary valve. Invaginating balloon trunks were added to stabilize the hyperdynamic hearts while accurately positioning the TAVR in the absence of an X-ray footprint.

## Materials and Methods

### Balloon-Expandable TAVR Stent for Bioprosthetic and Polymer Leaflets

The design of the TAVR stent was based on three principles: (1) continual stent-scallops for leaflet attachment that are crimpable and restored to their original shape upon balloon expansion; (2) self-elevating inter-commissural anchoring arms based on geometric changes due to plastic deformation occurring after crimping during deployment and (3) an hourglass shape with the waist seated in the annulus plane resulting in a concavity around the bulge of the crest of the muscular ventricular septum, easing the pressure on the crest (24, 25).

**Figure 1:**
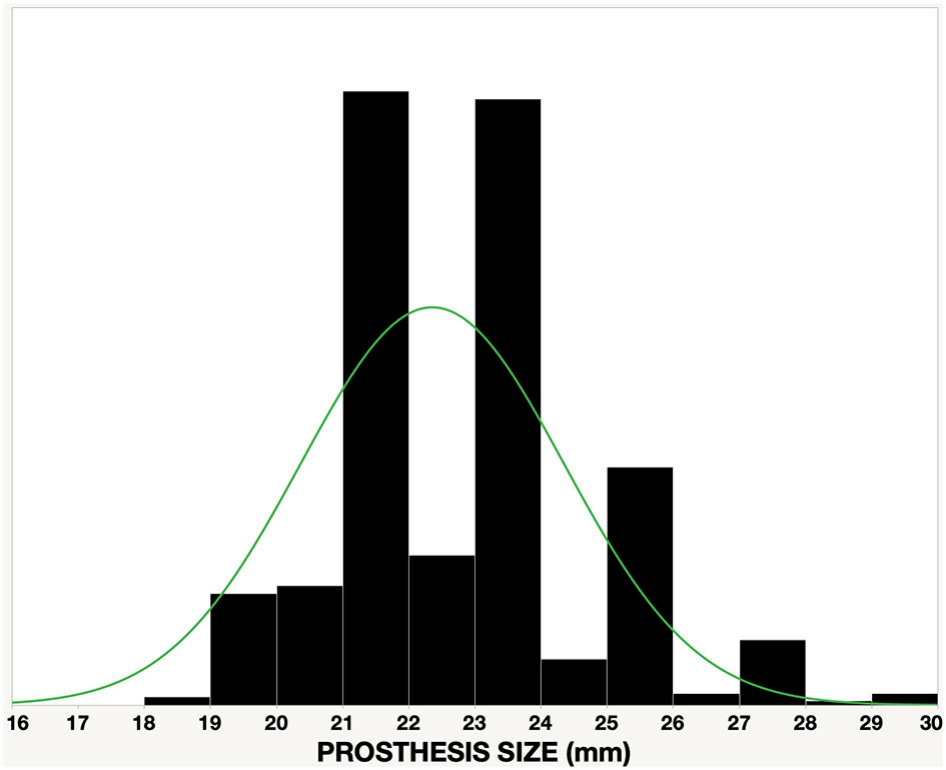
Size distribution of surgically implanted valves in a cohort of 350 patients with mainly rheumatic aortic regurgitation at Groote Schuur Hospital, University of Cape Town.

**Figure 2:**
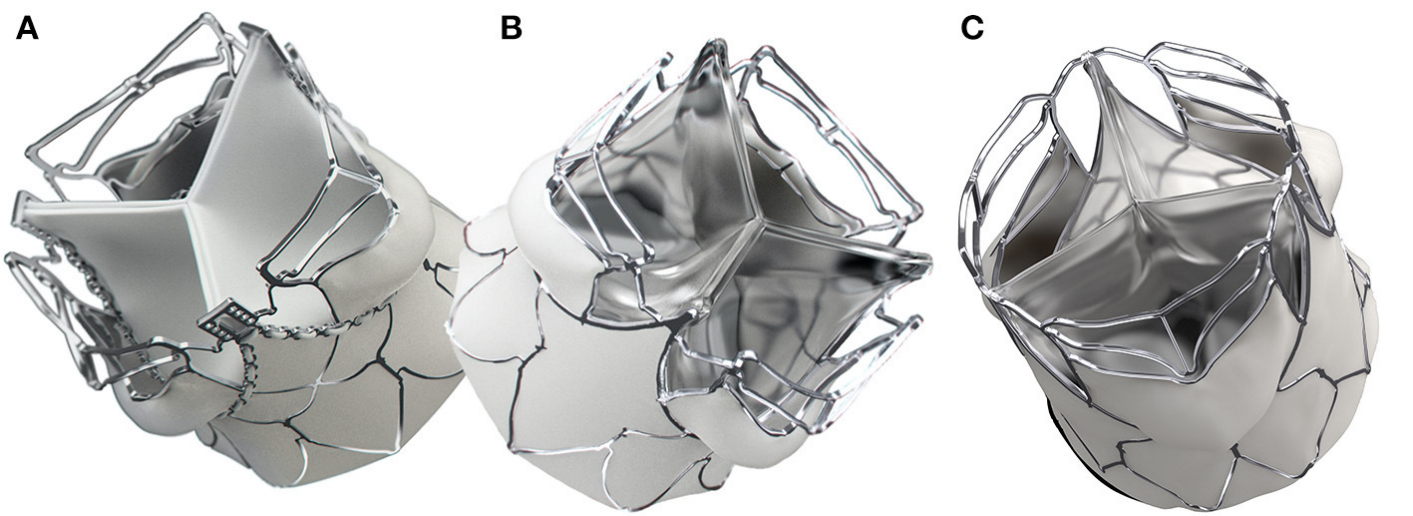
SAT balloon-expandable TAVR valve. The direct bonding to the MP35N-scallop allows an optimal attachment of polymer leaflets to the stent. Both the supra-annular anchorage arms and the spacer arms are structures that are self-elevating on the basis of plastic deformation. The first generation SAT TAVRs show minor differences between the bioprosthetic **(A)** and the polymer version **(B)**. The second generation “universal” stent **(C)** supports both bioprosthetic and polymer leaflets and allows crimp-diameters for trans-femoral access.

Stents of three sizes (Small “S,” Medium “M,” and Large “L” were cut from 23, 26, and 29 mm OD nickel-cobalt-chromium alloy MP35N tubes (Minitubes, Grenoble, France), respectively, using a Tube-Fiber Laser cutter (wavelength 900–1250 nm; StarCut; Rofin/Coherent Inc., Plymouth, MI, USA). Sizes were based on the size distribution of surgically implanted prostheses for rheumatic AR at the University of Cape Town (Figure 1). Scallop-struts were designed smooth to accommodate polymer (PU) leaflets and with stitching holes for bioprosthetic (BP) leaflets (Figures 2A,B).

ABAQUS (Dassault Systèmes Simulia Corp, Providence, Rhode Island, USA) was used for the Finite Element Analysis (FEA) assuming isotropic elasto-plastic materials. Stent fatigue was tested at 25 Hz for 400 million cycles at 6–8.5% compliance at ΔP 120 mmHg, 37°C (BDC Laboratories RDTL-0200-3600i, Wheat Ridge, CO, USA).

### Second Generation: “Universal” TAVR Stent

A prototype universal stent was developed for sizes M and L, providing one stent design for both BP and PU leaflets while reducing the crimp-size to allow both transapical (TA) and transfemoral (TF) delivery. The gap between two horizontal struts of the upper spacer-arm was increased to facilitate future coronary access in the unlikely event that the left coronary artery (LCA) was below the upper stent strut (Figure 2C).

### Valve Leaflets and Skirt

**Figure 3:**
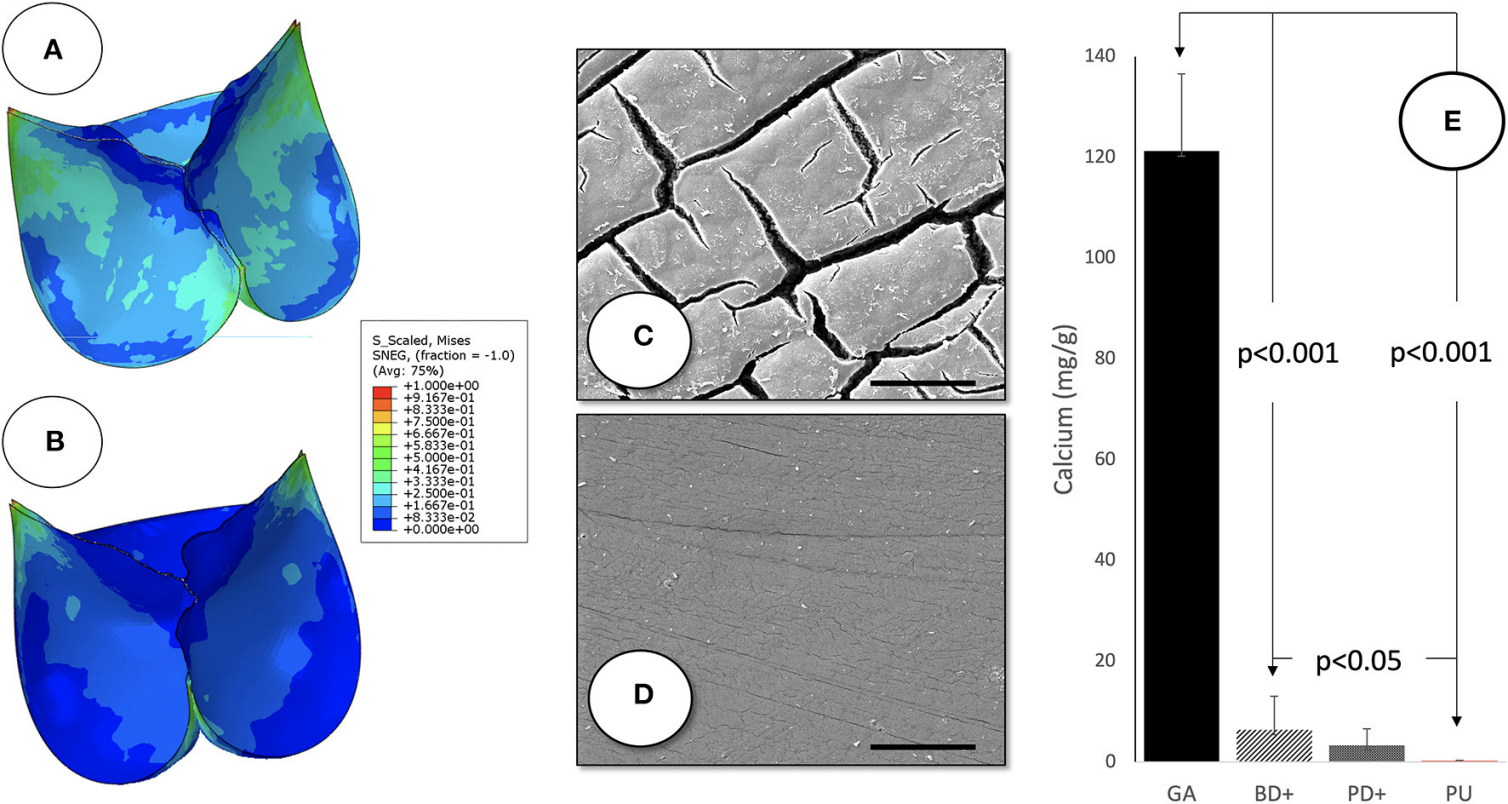
Leaflet characteristics of SAT TAVR: normalized Von Mises stress of the PU **(A)** and BP **(B)** leaflets in the closed position shown as a contour plot of FEA results. Scanning electron micrographs of 100% pre-strained polyurethane leaflet films after 25 weeks of subcutaneous implantation in long-Evans rats demonstrating the degradation resistance of the SAT Carbosil leaflets **(D)** compared to significant surface degradation visible on the Pellethane control samples **(C)** (5,000×; Scale bar = 10 μm). While calcification **(E)** was distinctly reduced in the decellularized, sandwich-crosslinked bioprosthetic leaflets **(D)** both in bovine (−95%; left) and porcine (−97% right) pericardium compared to the control group (GA), Calcium levels were almost undetectable in the group of pre-strained Carbosil samples.

The leaflet design was based on Bézier curves. Stresses were optimized using FEA on the basis of isotropic hyperelastic materials (Figure 3). Decellularized, sandwich-crosslinked Namibian bovine pericardium (porcine for the “universal” stent) was processed as previously described (26). The potential longevity of leaflets was assessed in the rat subcutaneous model. Leaflet discs were implanted into 5-week-old Long–Evans male rats for 6 weeks. Decellularized and sandwich-crosslinked bovine and porcine pericardium as well as Carbosil (100% pre-strained on Co-Cr lattice frames to simulate the contact of the leaflets with valve stents) were compared with standard 0.7% glutaraldehyde fixed bovine pericardium. Calcium contents were analyzed by Inductively Coupled Plasma Optical Emission Spectroscopy (ICP-OES; Spectro Arcos, Kleve, Germany) and expressed as μg/mg of dry mass. Pre-strained 25 week implants of Pellethane and carbosil film-strips (4 × 1 cm, 150 μm thick) were analyzed by scanning electron microscopy to visualize any degradation of the sample surface.

BP-leaflets were stitched onto the stent *via* suture holes using 5-0 Ticron sutures. PU valves were manufactured by a robotic arm in a combination spray process using different hardnesses of CarboSil (DSM Engineering Materials Inc., Evansville, IN, USA). Electrospun polymer skirts (thickness: 110–140 μm; pore size: 20–100 μm^2^) were externally heat-welded onto the stent.

### Dimensions During Deployment

BP and PU valves were assessed in conjunction with the SAT non-occlusive TA deployment system. “Universal” SAT TAVR were assessed using conventional deployment balloons for TF delivery. Since the different sizes of the system represent scaled versions of one basic design, detailed dimensional analyses were obtained for the M system. Dimensional changes during balloon inflation were recorded against increasing filling pressures at 1 bar increments. The diameters were measured at the level of the distal end of the stent, at the levels of maximal expansion of the top (“spacer”) and bottom (“supra-annular”) arms, at the narrowest waist (beneath the nadir of the scallops) and at the proximal end (“ventricular flare”). Stent recoil was assessed from the dimensions at each point with the balloon inflated and deflated.

### Fluid Dynamics, Crush-Force and Fatigue-Testing

Hydrodynamic testing to determine gradients (ΔP), effective orifice areas (EOA) and regurgitant fractions was performed in a pulse duplicator (Cardiac Output: 5 L/min; 37°C; 70 bpm; Stroke Volume: 32 ± 5 ml; Systolic Phase Duration of 35 ± 5%) (ViVitro Labs Inc; Victoria, BC, Canada). The radial crush force was determined by using a radial expansion tester (RX650 Radial Expansion Equipment, F033919 Head) and a conditioning chamber (Machine Solutions Inc. [MSI], AZ, USA). Accelerated durability testing was conducted using a BDC Laboratories VDT-3600i Valve Fatigue Tester (BDC Laboratories, Wheat Ridge, CO, USA) for up to 500 million fatigue cycles at 15 Hz in 0.9% saline (containing 0.2–1.5% Biguanide 20 bactericide) at 37°C. After every 50 million cycles, the valves were evaluated for structural damage and hydrodynamic testing.

### Non-occlusive TA Deployment System

The delivery device had four requirements: (1) a non-occlusive balloon; (2) tactile placement in and stabilization of hyper-dynamic hearts with unpinchable retractable feelers; (3) a back-flow valve that permits protracted delivery (4) and an atraumatic retrieval system.

The hollow-balloon was based on a helical tube held by a fine-meshed Nitinol frame. The locator/stabilizing arms were based on balloon tubes that could be retracted through invagination. The retrieval sheath rolls over the crimped valve and the distal end of the delivery device, thus eliminating shear between the sheath and the device. Both helical and trunk balloons use thin walled, high UTS polyethylene terephthalate (PET) (wall thickness: 25–33 μm) while the retrieval “rolling” sheath uses Nylon 12. End points of trunk-balloon optimization were the maintenance of stability in the extended state, torque resistance and prevention of buckling during retraction. Test systems were based on 3D printed jigs and fixtures, a force gauge (FG-6005SD, Lutron Electronic Enterprise Co., Ltd.), Torque Gauge (BTG26CN, Tohnichi Mfg. Co., Ltd.) and a tensile testing machine (Instron 5544, Instron® Norwood, MA United States).

Balloon performance testing established the rated burst pressure and the safety factor based on functionality or as guided by ISO 25539-1 using a custom-made burst pressure rig. This was also used to evaluate the ability of the balloons to withstand repeated cycles/inflations as guided by ISO 25539-1 and for specific creep testing to determine the safety factor before time dependent deformation affects functionality during use. Tensile (water bath, Instron 5544) and torsional tests were performed to evaluate the respective peak forces at each junction of incremental diameter increases as guided by ISO 10555-1. Radial force measurements (RX650 Radial Expansion Equipment, F033919 Head, Machine Solutions Inc. [MSI], AZ, USA) determined per unit length of the helical balloon were related to inflation diameters and pressures. Occlusiveness of the helical balloon in relation to filling pressures was established for the M-size system.

### Dilatation Balloon Catheter

A TF dilatation balloon catheter for aortic pre-dilatation was developed following the same non-occlusive principle as the deployment balloon but designed to minimize inflation and deflation times, thereby minimizing the occlusive phase in the absence of rapid ventricular pacing (RVP). Pressure gradients across the expanded balloon and radial forces were determined. A wall thickness of 14 μm (Nordson Medical) was chosen to minimize the catheter crossing profile while still maintaining device safety and performance. Simulated use and burst pressure, fatigue performance and tensile tests were carried out in accordance with ISO 10555-1 and ISO 10555-4.

### Simulated TAVR Placement and Anchorage Testing

To evaluate the ability to access, deploy and withdraw the devices, an ex vivo porcine heart (XH) loop system was used to simulate cardiac flow, using a software-controlled piston pump with variable stroke volume and heart rate. An adjustable atrial reservoir provided constant positive pressure to the atrium, a flow resistor adjusted the arterial pressure and a Windkessel provided shape-modulation of the pulse-wave and diastolic back-pressure (27). The deployment procedure was endoscopically visualized after insertion through the stump of the brachiocephalic artery. The mock-circulation was used in combination with a Philips angio-permissible C-Arm (Philips BV Pulsera mobile C-arm system, Philips Medical Systems, NL). The deployment process commenced with trunk-inflation in the ascending aorta (at 12 bar) and tactile trunk-location in the nadirs of the native leaflets. The TAVR was then deployed at 18 bar followed by the retrieval of the deployment system using the pressurized rolling-sheath. The correct position of the TAVR was confirmed through an oblique ventriculotomy. Pull-out resistance of the valve was tested with a Lutron FG-60055D force gauge.

A second test system used a Pulse Duplicator loop that simulates flow through a 3D printed annular ring, with a crush force of 3 N when expanded to the maximum waist diameter corresponding with the landing site for the trunks during the deployment process. This commenced with trunk-inflation above the ring (at 12 bar) and tactile trunk-location on the landing site, followed by retrieval through the pressurized rolling-sheath.

## Results

The BE TAVR system met all requirements for which it was developed: the expansion-linked shape-change of the stent resulted in the profile differences required for anchorage in non-calcified aortic roots; the scallop-design allowed for the direct, fatigue-resistant attachment of both elastomeric and bioprosthetic leaflets and the deployment device permitted root stabilization and tactile placement during uninterrupted cardiac output.

### Stents

The BP stents (S and M) were fatigue-tested to 400 million cycles; no strut breakage occurred. Radial Crush Force was 102.45 ± 4.63 N (S), 116.42 ± 4.20 N (M) and 115.00 ± 5.00 (L) for the BP stent and 124.47 ± 16.70 N (S), 103.04 ± 5.83 N (M) for the PU stent, respectively. Design modifications and strut width reductions with the goal of a “universal” stent for both TF and TA delivery only modestly reduced the crush resistance by 9.6% when compared to the BP stent (M).

### Leaflets and Skirt

FEA modeling of leaflets defined the extra length of the free edge to accommodate top-flaring of the stent. Decellularised, sandwich-crosslinked tissue showed markedly and significantly less calcification (6.4 ± 6.6 and 3.3 ± 3.2 μg/mg for bovine and porcine respectively) than GA-fixed non-decellularized pericardium (121.2 ± 15.3 μg/mg; *p* < 0.001). The polyurethane implants (Carbosil) showed practically no calcification at all (0.28 ± 0.07 μg/mg), significantly less than even the decellularised tissue (*p* < 0.05) (Figure 3). After 25 weeks of subcutaneous impantation the pre-strained Carbosil samples showed hardly any surface degradation while the Pellethane samples were visibly and heavily degraded on their surfaces.

**Figure 4:**
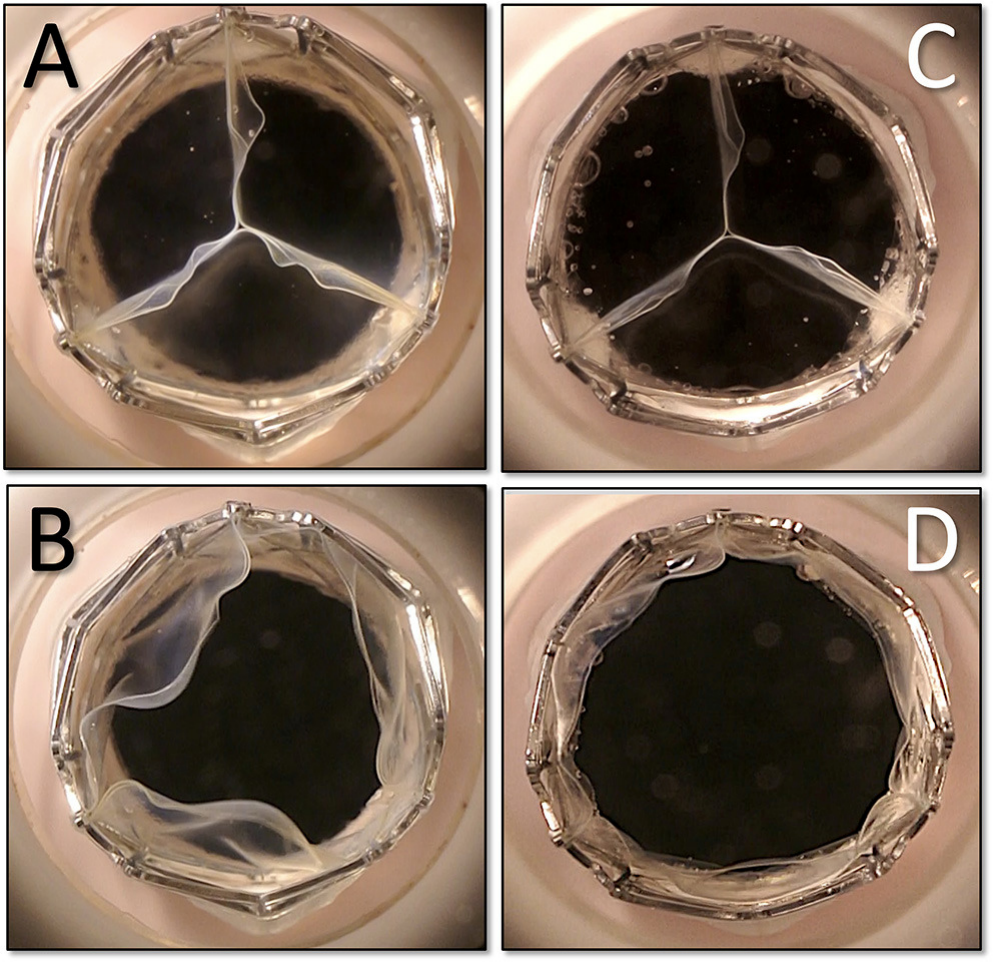
Fine-tuning of the thickness of the polymer leaflets in the pulse duplicator. With twice the cusp thickness on the left side **(A,B)** end-diastolic coaptation is identical **(A,C)** but endsystolic opening shows a more complete hinge-motion in the thinner leaflets **(D)** eliminating some areas with a potentially lower wash-out effect. The flaring of the top of the stent and the diameter increase is visibly compensated by the clam-shell design of the leaflets.

**Figure 5:**
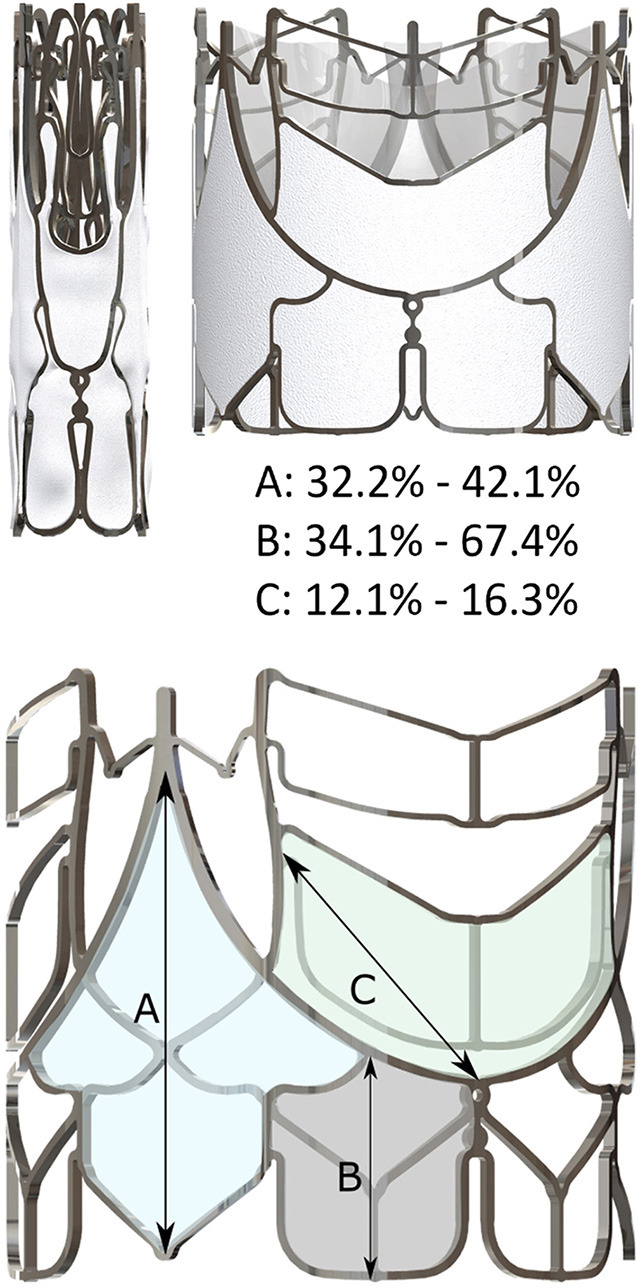
Crimping of a scalloped stent leads to distinct elongations of the skirt. Stretching is most pronounced along the indicated vectors **(A)** at the commissures; **(B)** at the infra-annular flare and **(C)** on the supra-annular arm.

**Figure 6:**
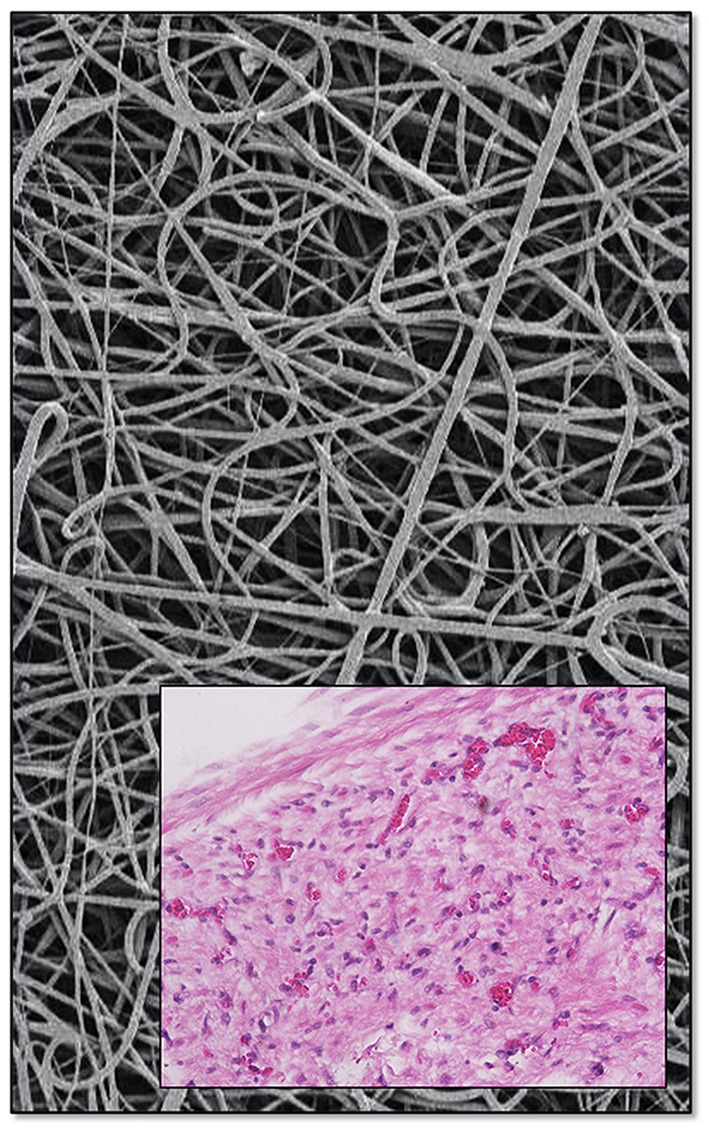
Skirts are electrospun from the same material used for the polymer leaflets. Porosity was shown to allow transmural capillarization [bottom, from (17) with permission].

Leaflet thickness was 340 ± 31 μm for BP and 150 ± 14 μm for PU leaflets. Both materials reached the predetermined 400 million cycles, neither leaflet type showing signs of macro-degradation such as delamination of the free leaflet edge. In the pulse duplicator, PU and BP leaflets showed equal fluid dynamics with excellent EOA, pressure gradients and regurgitation volumes (Table 1). For the universal stent, leaflet thickness of treated porcine pericardium was 137 ± 22 μm and that of PU leaflets was 52 ± 8 μm. While fatigue testing and regurgitation-fraction have not been finalized, fluid dynamics were improved (Table 1). Thinner polymer leaflets also led to reduced zones of incomplete hinging during systole (Figure 4). Across the three TAVR sizes, crimping was shown to expose the electrospun skirts to a strain of up to 67%. No tearing or detachment from the welding lines occurred in the crimping and re-expansion tests (Figure 5). Skirt permeability was shown to be 1,616 ± 1,344 ml/min/cm^2^ sufficient to allow transmural capillary ingrowth (Figure 6)(17). The lowest point of the skirt was always the lowest point of the void between commissural posts and stent arms (Figures 2, 5).

**Table 1:** Dimensional and hemodynamic characteristics of SAT TAVR [transapical (TA) and universal (Univ)] comparing bioprosthetic (BP) with polymer (PU) leaflets, in combination with the non-occlusive trans-apical delivery system (TA-DD) or a conventional trans-femoral (TF) delivery balloon.

		**BP/TA**	**PU/TA**	**BP/Univ**.	**PU/Univ**.
Non-occlusive DD	Stent top	27.03 ± 0.17	27.19 ± 0.39	25.32 ± 0.08	26.52 ± 0.33
(TA delivery) (diameter at 18 bar in mm)	Spacer arm	29.45 ± 0.19	28.08 ± 0.53	29.31 ± 0.56	29.39 ± 0.11
	Supra-annular arm	30.14 ± 0.13	30.69 ± 0.54	29.72 ± 0.81	30.69 ± 0.25
	Nadir/landing zone	24.42 ± 0.20	24.50 ± 0.35	24.65 ± 0.12	24.67 ± 0.16
	Bottom flare	29.54 ± 0.49	28.18 ± 0.98	28.16 ± 0.06	28.48 ± 0.26
	EOA/cm^2^ (ISO limit: 1.58)	2.39 ± 0.03	2.34 ± 0.09	2.43 ± 0.04	2.38 ± 0.10
	ΔP (mm/Hg)	6.17 ± 0.14	6.72 ± 0.20	5.93 ± 0.13	6.40 ± 0.35
	Regurgitant fraction (%)	7.60 ± 0.77	7.25 ± 0.60	11.97 ± 0.35	6.92 ± 0.76
	Crimp diameter (mm)	9.17 ± 0.06	9.24 ± 0.47	8.94 ± 0.09	9.21 ± 0.12
Conventional TAVR balloon	Stent top			25.02 ± 0.30	25.78 ± 0.17
(TF delivery) (Diameter at 5 bar)	Spacer arm			28.30 ± 0.53	28.19 ± 0.41
	Supra-annular arm			31.49 ± 0.28	31.55 ± 0.67
	Nadir/landing zone			24.57 ± 0.09	24.46 ± 0.21
	Bottom flare			29.27 ± 0.26	29.52 ± 0.16
	EOA/cm^2^ (ISO limit: 1.58)			2.41 ± 0.01	2.31 ± 0.11
	ΔP (mm/Hg)			6.03 ± 0.01	6.59 ± 0.47
	Regurgitant fraction (%)			10.65 ± 0.15	6.25 ± 0.97
	Crimp diameter (mm)			6.45 ± 0.11	6.55 ± 0.06

*Only the Universal design is suitable for both TA and TF delivery (measurements in millimeters)*.

### TAVR Dimensions and Deployment Dynamics

Crimped onto a conventional 26 mm trans-femoral TAVR deployment balloon, the diameters of the M-size BP and PU-TAVR measured 6.45 ± 0.11 mm and 6.55 ± 0.06 mm, respectively, compared with 9.17 ± 0.06 mm and 9.24 ± 0.47 mm when crimped onto the 26 mm non-occlusive trans-apical SAT hollow-balloon. Dimensions of TAVR valves (stents plus leafets and skirts) are listed in Table 1.

**Figure 7:**
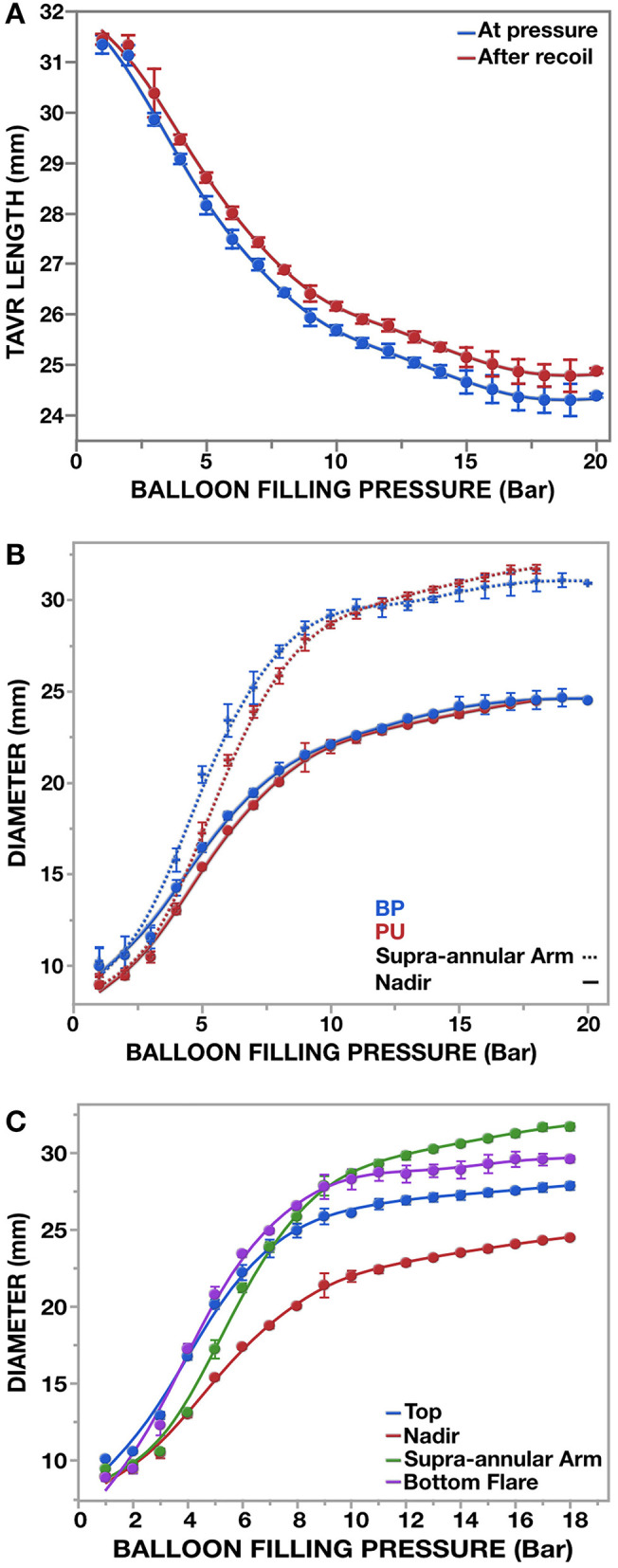
Outer dimensions of (M)-size pericardial (BP; *n* = 3) and polymer (PU; *n* = 3) SAT valves during expansion with a 26 mm non-occlusive SAT delivery device (*n* = 6). Measurements were taken at inflation increments of the balloon of 1 bar. **(A)** Overall shortening of the crimped valve during deployment. **(B)** A main feature of the SAT TAVR is that anchoring arms made of a non-shape memory alloy elevate on the basis of plastic deformation during expansion. The resulting diameter difference between waist and anchoring arms is already fully developed when the valve has only reached 60% of its maximum diameter. **(C)** With the supra-anular arms and the bottom flare having the biggest diameter difference to the “waist (nadir),” anchorage in compliant aortic roots is secured in both directions.

Longitudinal shortening of the valve during expansion after crimping was initially distinct but then flattened out non-linearly (Figure 7A). At 14 bar inflation pressure the crimped valves shortened from 31.53 ± 0.02 mm for BP and 31.34 ± 0.01 mm for PU to a post-deployment length of 24.87 ± 0.31 mm and 24.69 ± 0.02 mm respectively. Further pressure-increases to 18 bar had little additional effect on shortening.

**Figure 8:**
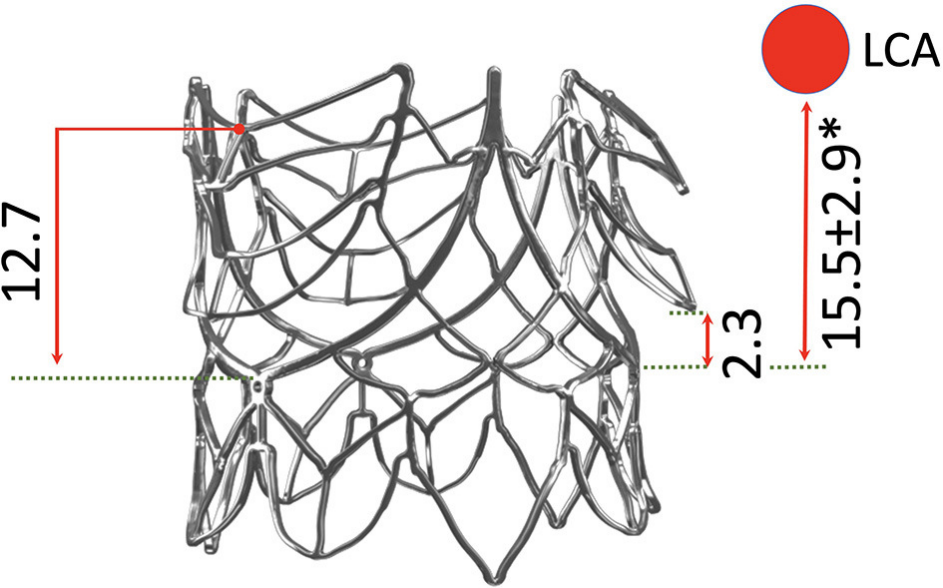
Profile view of the SAT TAVR stent relating key parts to the annular plain and showing the extent of arm-elevation achieved purely by the expansion force of the balloon. The average distance of the left coronary ostium (LCA) is shown in relation to the stent (*) (28–30).

**Figure 9:**
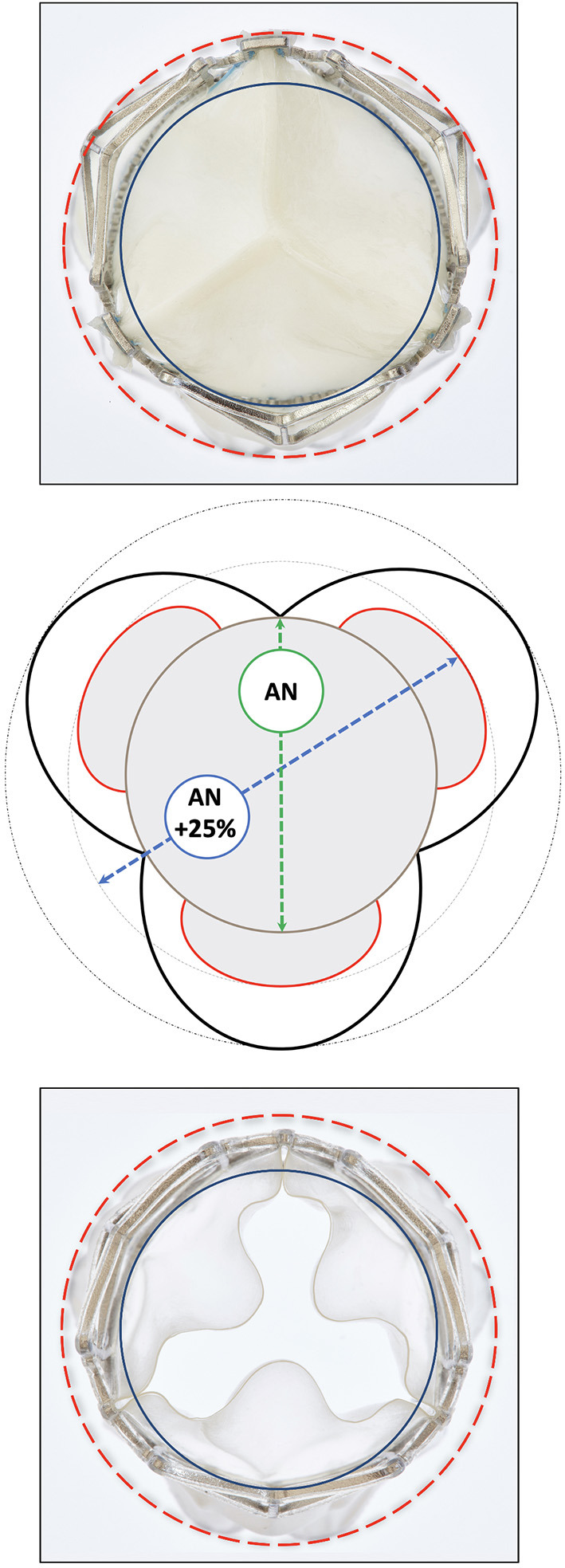
Top view of a deployed SAT bioprosthetic (top) and polymer (bottom) TAVR showing the radius of the supra-annular anchoring-arms (red) *vis a vis* that at the waist level corresponding with the annular landing zone (blue). The schematic drawing (middle) shows that the supra-annular diameter is 25% bigger than the annular landing zone of the TAVR firmly securing anchorage (pull-out resistance >23 N) even in the absence of calcification. There is still ample space between the arms and the sinus wall in average sinuses of Valsalva.

Radial expansion favored the wing-structures (Figures 7B,C), which, together with flaring of the bottom end of the stent, resulted in an hourglass-shape (Figure 8). The waist (corresponding with the landing zone) was in immediate proximity beneath the nadir of the scallops, right below the supra-annular anchoring arms. When fully deployed, a circle defined by the outermost points of the anchoring arms exceeded the outer diameter at the waist level by 26.5 ± 0.4/29.5 ± 0.6% (Figures 7–9; Table 1) introducing the distinct anchoring principle of the mid-portion of an hourglass against the limited distensibility of the annulus. The elevation of the stent arms was already fully developed at 6 bar inflation pressure [diameter-difference 21.9% BP/ 16.4% PU] when the OD of the stent-waist was only 18.21 ± 0.22 mm (BP) and 17.40 ± 0.12 mm (PU), respectively (Figures 7B,C). While the waist increased from 20.69 ± 0.42 mm/20.04 ± 0.12 mm to 23.77 ± 0.09 mm/23.51 ± 0.09 mm between 8 and 14 bar it only minimally increased further from 14 to 18 bar (24.54 ± 0.50 mm/24.48 ± 0.07 mm). From 6 bar onwards, corresponding with two thirds of the diameter at full expansion, the OD of the anchoring arms exceeded that of the waist by one quarter. Incremental inflations and deflations led to only 0.52 mm bigger waist diameter at 18 bar compared to single inflation. Over the entire range of non-occlusive inflation pressures, average post-deployment recoil was less than 2.47%/3.17% for the waist and 5.03%/6.29% for the wings of BP and PU valves.

### Non-occlusive Deployment System

**Figure 10:**
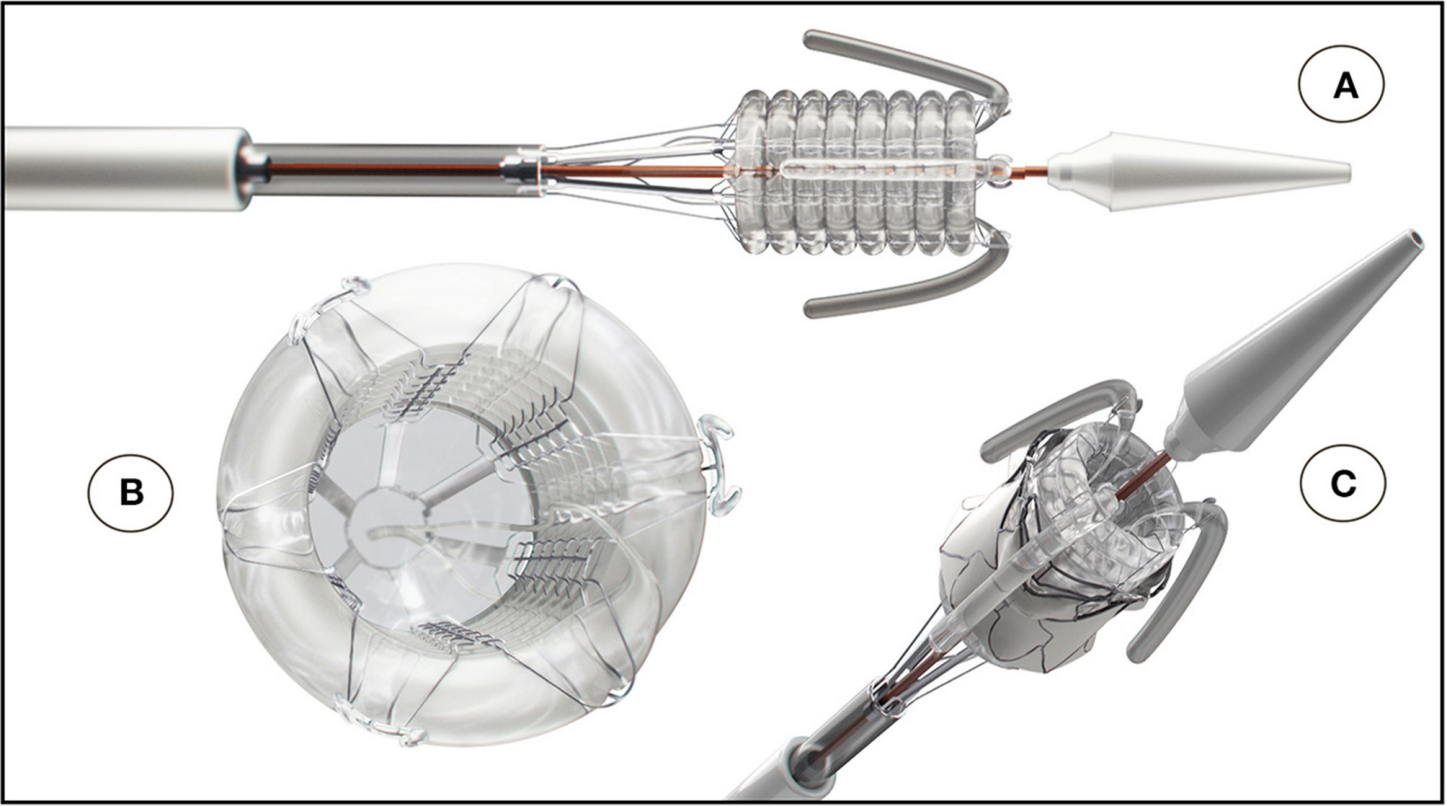
The SAT non-occlusive balloon-delivery system consisting of a helical balloon is prevented from toppling by a Nitinol frame; **(B)** positioning- and stabilizer-trunks that are invaginating upon retraction; **(A,C)** a back-flow valve and **(B)** a pressurized rolling sleeve for device retrieval [**(A)** Reproduced from (31) with permission].

**Figure 11:**
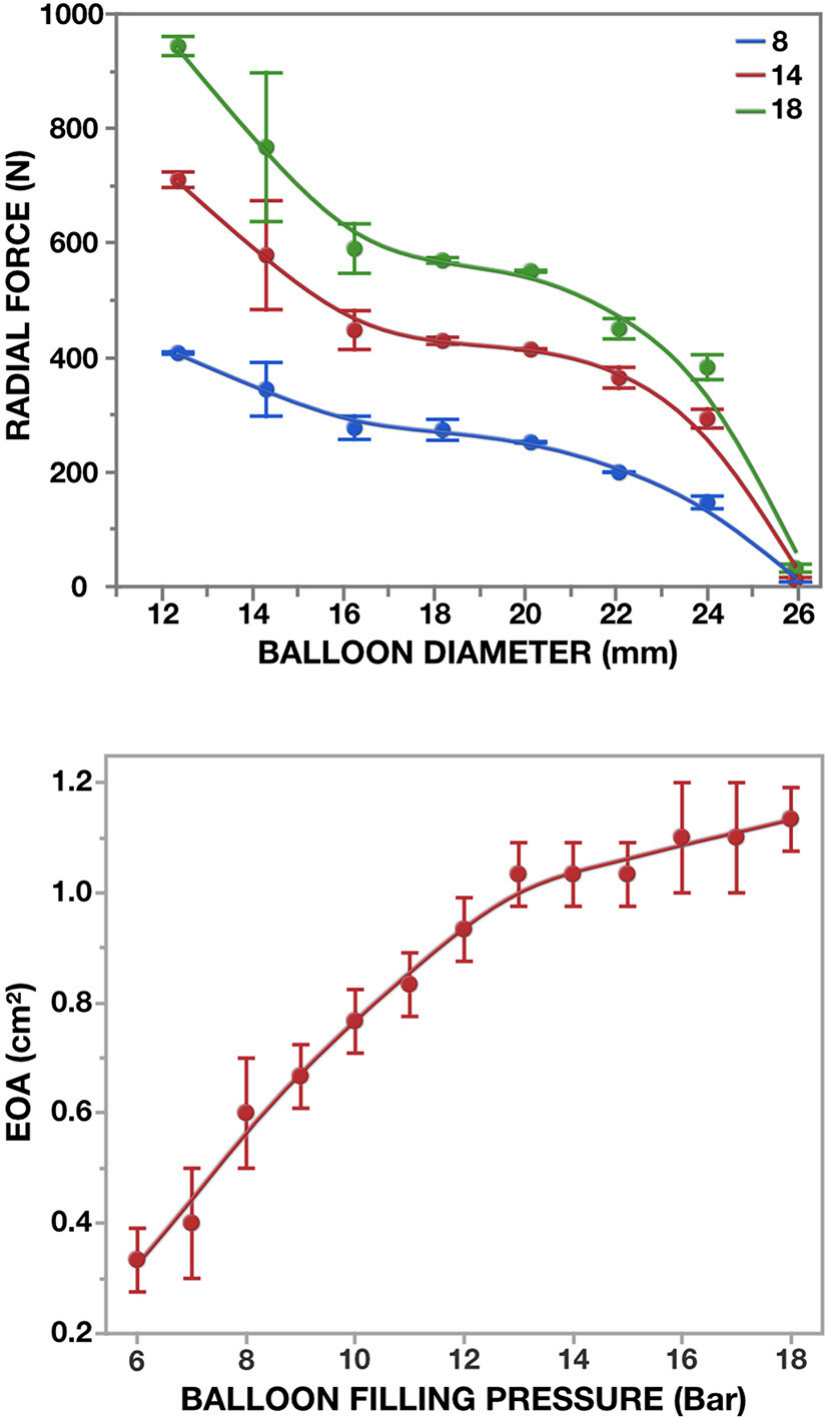
Radial force of the 26 mm non-occlusive delivery device for [M]-size TAVR. Three different inflation pressures were blotted against balloon-diameters (top): 8 bar (onset of non-occlusiveness); 14 and 18 bar (*n* = 3 × 3). In the pulse duplicator, effective orifice areas were determined against restricted balloon inflation (bottom).

Rated burst pressures were 18.4/18.3/5.6 bar for the trunk balloon, helical balloon and retrieval balloon, respectively. Each inflation was safely repeatable with a fatigue factor of 2 and a creep safety factor of 3/6/5. Figure 10 shows the main components. During the tensile and torsional tests, each bond exceeded the predetermined tensile and torsional load required to withstand component embolism. Radial force measurements of the helical balloon are shown in Figure 11A and the EOA during deployment in relation to filling pressure in Figure 11B. In the XH tests, the supra-annular arms anchored the stent on the rim of the annulus sufficiently below the LCA ostium (Figure 8) without diminished coronary outflow or obstruction.

### Simulated Use Testing

Endoscopic visualization during simulated *ex-vivo* placement (n = 21 S) and (n = 30 M) of BP and PU valves confirmed first attempt engagement of all three leaflets in 81 and 77 % respectively. The native commissure to TAVR commissure was rotationally in congruent alignment in 67 and 87 %. The anchoring arms were tightly snuggling supra-annularly onto the annulus in 76 and 90%. Post implant pull-out force was 23.50 ± 2.52 N for both stent sizes.

### Dilatation Balloon Catheter

**Figure 12:**
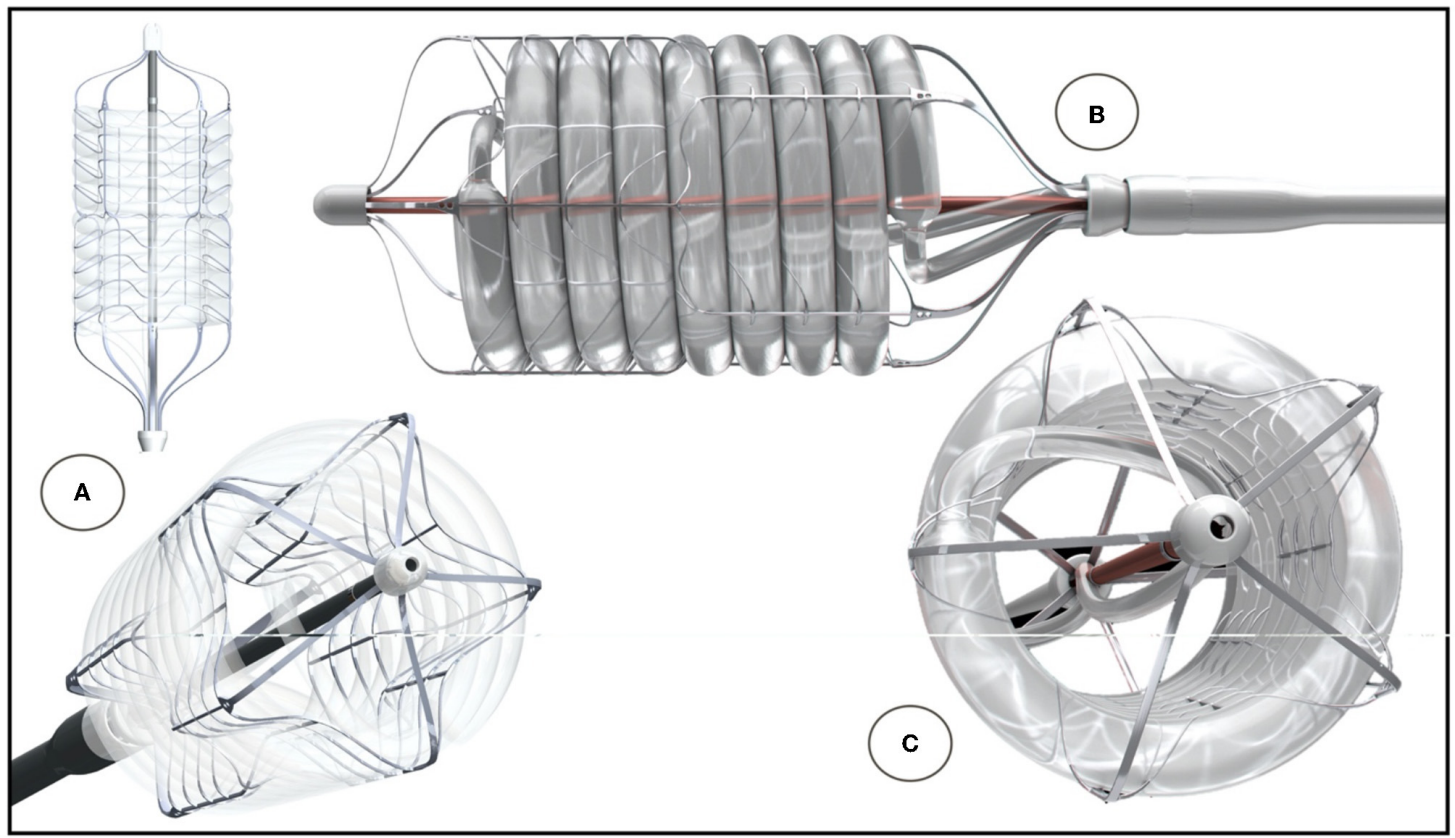
SAT helical dilatation balloon. The helix is prevented from toppling by a fine-meshed Nitinol frame **(C)**. A directional change at mid-level allows mirror-imaging of the proximal and distal ends **(A)**. Profile of combined helical balloon and support frame **(B)**. Dual inflation from both sides and relatively large feeding lines allow rapid inflation **(C)**.

**Figure 13:**
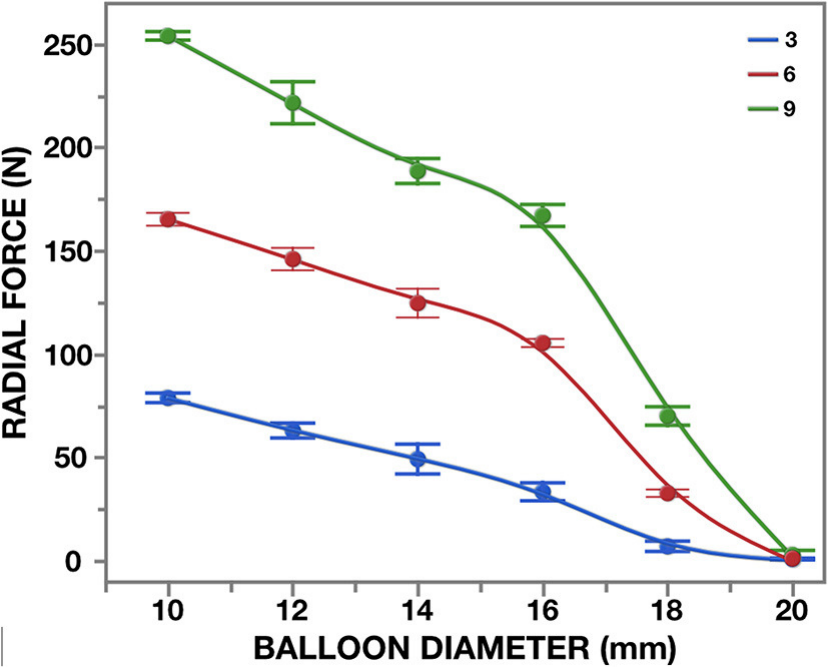
Radial force of the 20 mm dilatation balloon at three different inflation pressures (in Bar).

The dilatation balloon catheter is shown in Figure 12. Safety was satisfactorily demonstrated for the 20 mm balloon: each sample was inflated at least 10 times to RBP of 9 bar without device failure. Inflation time ranged between 0.27 and 0.35 s between different users and deflation time was 2.74 ± 0.58 s. When inflated, the mean gradient across the device during physiological pulse-duplicator generated flow was 32.68 ± 7.64 mmHg. Inflation-pressure dependent radial force measurements of the 20 mm balloon catheter are shown in Figure 13.

## Discussion

Utilizing differential plastic deformation rather than shape memory, with a dimensionally stable scallop design and the radial force of helical balloons, we have developed a fundamentally new concept of balloon-expandable TAVR that may greatly broaden its clinical use.

### Balloon-Expandable (BE) vs. Self Expanding (SE) TAVR and Permanent Pacemaker (PPM)

For almost two decades it has been presumed that SE TAVRs would supplant BE systems, even though the latter remained the unchallenged preference of clinicians. After 20 years experience, two-thirds of implanted TAVR in the USA are BE (32). In Europe, SE TAVR devices together account for less than half of the market (32) and the situation is even more extreme in Japan where three quarters of TAVRs are BE (33). The perception that the future belongs to SE TAVR is self-perpetuating, which is astonishing with respect to clinical realities. Huge cohort studies repeatedly showed key advantages of BE (33–37) the most prominent being the persistently higher need for a permanent pacemaker (PPM) with SE devices (33–35, 38–42), even if used for valve-in-valve procedures (43). The acceptance of these drawbacks by many clinicians in HICs is partly because the implantation of a pacemaker is affordable. This higher need for PPMs in SE devices can be related to their radial force profiles (44). SE valves exert less radial force than BE counterparts during deployment and therefore result in a higher degree of PVLs (41) and initial micro-dislodgement (45). However, they maintain a relatively high radial force beyond their nominal diameter while that of BE valves is limited to the diameter at deployment. Therefore, once a BE TAVR has healed in, the pressure on the conduction system has eased, whereas SE TAVRs continue to push against the surrounding tissue. Use of a shallower implantation depth (46) and non-flaring designs (47) have attempted to mitigate this, but recent studies confirmed continual contact pressure independent of implant depth as the key predictor for conduction abnormalities with SE TAVR (48). Moreover, as the PARTNER 3 trial showed, the disadvantage of a larger crimping diameter of BE systems has been minimized with expandable sheaths and when extending TAVR to low-risk patients (3).

Another perceived advantage of SE systems is their ability to be re-sheathed during deployment. Although appealing, this does not solve a general problem of transcatheter implantations but one that specifically concerns SE TAVR. The rate of re-sheating maneuvers required was 24% with the Portico system (47) and 23% in the Evolut-R US registry (49) but in modern comparative studies hardly any of the patients receiving BE TAVR would have required it (50). While the combination of affluence, local experience and skill-profiles may make a case for either BE or SE in HICs, the avoidance of expensive adjunct procedures would make BE devices more affordable and suitable for the vast number of potential patients in LMICs.

### TAVR Needs in AR Patients: More Than a Fringe Group

The specific needs of MICs, including their high proportion of relatively young patients with AR and often the lower level of skills and equipment available, has slowly begun to challenge the unwavering bet on the unlimited growth trajectory of Western products in these regions (9, 10, 20, 31, 51). A majority of symptomatic patients in countries such as China, India, Brazil and South Africa require AVR for rheumatic regurgitation (9, 10, 13, 31, 52). While often concealed by the fact that the leading heart centers in these countries disproportionally cater for an aging urban population that partially mirrors HIC pathologies these data have long been available. In a study from Shanghai's Zhongshan Hospital that assessed 315,884 patients with moderate to severe aortic valve disease, only 27% were above 65 years of age and even in this subgroup AR outweighed AS by a factor three (13). Shanghai's Changhai hospital confirmed that the proportion of patients undergoing SAVR was significantly higher for AR than AS (11). The 2020 up-date of the “Chinese Expert Consensus on TAVR” confirms the high proportion of rheumatic etiology in Chinese patients with aortic valve disease (53).

Clearly, TAVR suitable for pure, non-calcific AR in younger patients would therefore not only be for a fringe-indication but of relevance for a dominant pathology in MICs (9, 10, 15, 31). Recent publications on the use of TAVR for RHD can be regarded as important harbingers of this development (14–17, 31) particularly since TAVR was shown to have a profound, durable impact on heart remodeling in patients with severe AR. Within the first 3 days, a significant decrease in LV end-diastolic pressure, a significant reduction in LV size and mass index (54) and a sharp reduction in systolic pulmonary arterial pressure were seen (55), confirming that in patients undergoing valve replacement for severe AR, cardiac function often recovers faster than in AS. In many areas with a high incidence of RHD, patients are breadwinners for extended families, so that symptomatic relief would critically affect livelihoods even in the absence of significantly extended life expectancy. Even then, minimizing the ischaemic myocardial injury associated with open heart surgery (OHS) by using trans-catheter procedures would increase the likelihood of remodeling, and thus of a longer life expectancy.

The scope for TAVR is also changing in HICs. According to the Euro Heart Survey (56) the treatment of severe pure AR clearly represents an unmet clinical need. For this reason, TAVR is being increasingly performed “off label” for pure AR in patients excluded by surgery (18). Already, TAVR patients transapically treated for AR had better in-hospital outcome compared with SAVR patients (39, 57). With TAVR increasingly being considered for patients with bicuspid aortic valves (58) the demand for devices suitable for AR will also grow, since 10–15% of these patients need AVR for pure AR (59). This particular indication would create an overlap between HICs and MICs as patients with such valves are prevalent in China (11). It is therefore foreseeable that TAVR designs will need to cater for both (57) AS and AR in order to have a true global appeal.

### Leaflet Durability: The Next Horizon for Both AR and AS Patients

The need for improved valve durability in younger patients is growing globally. Currently, TAVRs are largely restricted to patients in their seventies and older. As long as TAVR was confined to high risk patients, the vast majority of patients fell into this age category. This has changed with the approval of TAVR for lower risk patients (3, 41) but as these patients are usually younger many do not qualify for a TAVR. Therefore while “low operative risk” is now treatable by TAVR, “younger age” becomes the new reason for not qualifying. When all AVRs were performed through conventional cardiac surgery, there were no procedural choices, just formulaic decision of mechanical valves for the young or tissue valves for the old. The advent of TAVR for all risk categories has turned this situation into an exclusive privilege for some: non-invasive TAVR reserved for elderly patients while the need for a durable mechanical prosthesis still condemns young patients to open heart surgery.

In MICs the situation is even more extreme. Since RHD patients are often in their early fourties at the time of surgery (31, 60, 61) valve durability would need to be markedly improved to permit a trans-catheter approach. Since access to open heart surgery and post-implant control of anticoagulation is often severely limited, simple transcatheter approaches would provide hope for many (9, 10, 15, 31). Therefore, the highest bar for leaflet longevity is defined by the needs of patients with RHD in MICs. Achieving this would also profoundly address the needs of patients with degenerative AS in HICs who are currently deemed too young for a tissue valve and therefore for TAVR (62). Recent developments suggest that such improved leaflet durability is a real possibility as both major degeneration modes of soft-leaflet materials have been identified and successfully addressed: remnant immunogenicity in bioprosthetic materials (63) and biodegradation in polymers (28). The SAT TAVR implemented the most recent features in both its pericardial and polymeric leaflets. For the BP version the leaflets consist of decellularized, sandwich-crosslinked pericardium that completely suppressed calcific degeneration both in the rat (26) and the sheep model (17). At the same time, degradation-resistant polymers (64) promise to be a realistic alternative with potentially significantly longer lasting soft-leaflet heart valves. Recent first-in-man studies with polymeric surgical valves (65) have strengthened the belief that polymer leaflets represent an important opportunity for heart valve technology. Therefore, the SAT TAVR stent was specifically designed to provide optimal support for polymer cusps. The choice of Carbosil^®^ for the leaflets was based on excellent biostability (66) combined with mechanical properties, and wide clinical use in other high-performance applications such as drug eluting stents (67). Carbosil 2080A TSiPCU is a segmented triblock polyurethane which combines siloxane segments for biostability and carbonate segments for processability and toughness. Its excellent biostability has been confirmed both *in-vitro* (68, 69) and *in-vivo* (70). *In-vitro* experiments under both hydrolytic and oxidative conditions showed preservation of material properties in contrast to earlier generations of polyurethanes (71–73). Moreover, the surface properties of Carbosil also provided excellent biocompatibility, including haemocompatibilty (74–76). It does not induce conformational changes in any attached fibrinogen thereby preventing triggering the coagulation cascade and subsequent thrombus formation (77, 78). Moreover, the low intrinsic capacity of segmented polyurethane leaflets to calcify *in vivo* (79) has been confirmed by our results. Both the fatigue resistance with leaflets as thin as 50μm and the excellent preliminary *in-vivo* performance vindicate this material choice.

### Leaflet Thrombus and Valve Design

While leaflet durability is a prerequisite for the longevity of heart valves in younger patients, valve thrombogenicity and the potential consequences of subsequent leaflet immobilization and embolization (80) may also threaten valve endurance, particularly in the absence of anti-coagulation (81). Subclinical thrombus formation on valve leaflets following TAVR is increasingly recognized (82) ranging from 4 to 40% after 1 year (83). The most plausible explanation for this phenomenon is the largely absent vortex formation due to the small neo-sinuses between the prosthetic leaflets and the displaced diseased native leaflet or skirt, leading to increased blood stasis (84). Vortices generated by the sinuses of Valsalva during early systole and persisting into early diastole play a crucial role in reducing thrombus formation on the outflow-side of native aortic valves (82, 85, 86). Increasing the neo-sinus size by higher deployment of conventional TAVR was shown to reduce the stagnation zone seven-fold (87) but such a higher implantation level would need to be balanced with the risk of coronary occlusion (82).

**Figure 14:**
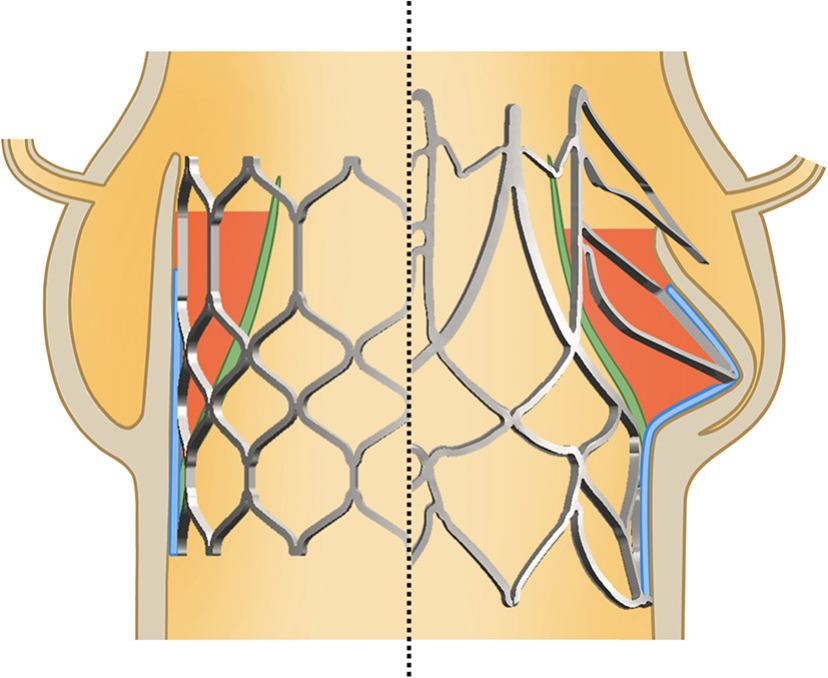
Difference between a conventional cylindrical TAVR and the SAT design: by skirting the supra-annular anchorage arms a spaceous neo-sinus is created between skirt (blue) and prosthesis leaflet (green) intended to facilitate sufficient vortex formation for the prevention of thrombus. This is opposed by the relatively narrow space of the neo-sinuses in cylindrical designs.

In the SAT valve the external skirt follows the shape of the supra-annular arms, creating spacious neo-sinuses. Furthermore, the direct insertion of the prosthetic leaflets into the scallops of the stent avoids spatial separation between the TAVR leaflet and the neo-sinus, creating a continuous physiologically shaped space intended to facilitate sufficient vortex formation (Figure 14).

At the same time, leaflet mobility and the degree of bending at the hinges of the leaflet insertion (Figure 4) add to the elimination of low-vortex zones. Our ability to produce thin, mechanically durable polymer leaflets has validated this concept in the challenging pig model, where thrombus formations previously seen at the nadir of thicker leaflets could be avoided by reducing their thickness.

Ingrowth-permissible skirt porosity further reduces the likelihood of thrombus formation in the neo-sinuses, as confined non-porous intra-vascular spaces increase that risk. Although poorly understood, non-porous vascular prostheses have a distinctly higher thrombosis rate than porous ones (88). Also, while most TAVR publications refer to delicate and functional endothelialisation on explanted leaflets in animal models (89), the actual tissue outgrowth onto the leaflet surfaces in humans consists of fibrotic transanastomotic pannus tissue (90). This pseudo-neointima with its “endothelial-like” surface layer does not have the non-thrombogenic properties of true endothelium and is likely to eventually consolidate hypoattenuated leaflet thickening (HALT) seen in TAVR (91). As described by Berger et al. (90) as early as in the 1960s this tissue response represents the typical prosthetic fibrosis-mode in humans and is initiated by the deposition of surface thrombi. While diminished vortex formation explains the fluid-dynamic trigger for these thrombi, the absence of a functional endothelium allows platelet aggregation and fibrinogen conversion to unfold uninhibited. Recent experimental work indicates that transmural capillarization may compensate for the deranged trans-anastomotic neo-intima outgrowth typically seen in humans (88, 92). In contrast to the densely woven PET and PTFE skirts of contemporary TAVR, the electrospun SAT skirt provides sufficient porosity for this healing mode (17, 88, 92). Therefore, the successful capillary ingrowth seen across the entire skirt thickness in long-term sheep implants (17) encourages the belief that the SAT TAVR valve may allow functional endothelialization in patients.

### A Balloon-Expandable TAVR for Non-calcified AR

The second main focus of SAT was to develop the first BE TAVR that also caters for non-calcified pure AR, the predominant pathology in RHD patients (9, 10, 13, 31, 52) which is also a prevalent occurrence in other pathologies including bicuspid aortic valves (59). Two major challenges face the use of TAVR in pure AR: anchorage in the absence of calcification and correct positioning in cases of excessive stroke volumes. Lack of calcium in the annulus and leaflets makes delineation of the annular plane difficult, requiring greater contrast exposure with a potentially elevated risk of acute kidney injury (93). Moreover, this lack of calcium conglomerates for anchoring requires considerable oversizing in conventional TAVR (to prevent migration or embolization) leading to a higher risk of pacemaker implantation (94) and annular rupture (93).

Of the SE valves which address anchorage in the absence of calcification (JenaValve, J-Valve, the discontinued Engager and Symetis/Boston Scientific Acurate) the first three featured supra-cuspal arms resting outside the leaflets in the sinuses. In contrast, the Acurate valve anchors with supra-annular arms on the ventricular side of the leaflets. Accordingly, the embolization rate of the Acurate TAVR was negligible in a “pure AR” study while conventional SE valves had a 50–58% embolization rate if they were <10% oversized but still 31–60% if they were >20% larger (95). Others have recommended oversizing at 15–25% or more for conventional SE devices or using up to 3 ml additional balloon-inflation volume for BE valves in AR patients (96). Alternative dedicated TAVR systems for AR improved outcomes but still had higher than acceptable procedural complications and mortality rates (18). While a dedicated SE-TAVR system for AR such as the JenaValve prevented embolization into the ventricle it still had the typical shortcomings of SE TAVR, with almost 10% needing a second valve implantation and 16% requiring a PPM (18). In two pure AR studies with the “Acurate Neo” valve, embolization could be prevented with moderate annular oversizing of 9% (54) but a PPM was still required in 15% of cases. This acceptable performance in AR was offset by failure to achieve non-inferiority with the Sapien 3 in patients with AS due to higher rates of severe PVL (97).

The SAT TAVR uses a supra-annular anchoring system in conjunction with a BE concept. Boston Scientific's Acurate is an SE TAVR that shares the principle of supra-annular anchorage. Both rely on a stent profile that prevents slippage through the annulus with an immediate supra-annular diameter exceeding that at the annular level by >20% resulting in a pull-out resistance of >23 N. However, while the supra-annular stent-expansion is only mildly wider in the SAT valve than in the “Acurate Neo” (26 vs. 20%), the difference in infra-annular flare is distinct (20 vs. 12%) (54). Since a significant proportion of TAVR embolization in patients with native valve AR is due to a forward dislodgement into the aorta (95) the inferior flare is crucial. In a TAVR study in patients with pure AR using predominantly Core- and Evolut-Valves, modest undersizing led to 50% of the embolizations occurring in antegrade direction. Even with oversizing, every 5th dislodged valve still did so toward the aorta (95).

In the SAT valve the infra-valvular flare is combined with a concavity intended to “contour-snuggle” along the septum beyond the muscular crest, even in a sigmoid septum. This combination of the restricted expansion of a BE stent with an anatomy-following shape resulted in the absence of conduction disturbances in 263 consecutive pig and sheep implants. This complete avoidance of heart block in two different animal models in spite of a distinct flare and an implantation depth that was previously identified as a risk factor for conduction damage (46, 98) supports the argument that it is the continual contact pressure exerted beyond the intended diameter by SE stents (48) rather than implantation depth or flaring that increases the risk of needing a PPM.

Most importantly, the plastic deformation of the stent during expansion does not only occur at full deployment but creates the distinct profile that anchors the valve from two thirds of the nominal diameter onwards (Figure 7) allowing measured sizing that further reduces the risk of conduction disturbances.

### Mitral Valve, Skirt and Coronaries

The mildly deeper implantation depth of the SAT TAVR may give rise to concerns regarding the mitral valve. While injuries to the anterior mitral leaflet are extremely rare, they have happened in TAVR valves with relatively sharp crowns (99). In contrast, the SAT stent has predominantly flat, round crowns; furthermore, modeling studies indicate that, counterintuitively, a lower stent position may have a lower risk for mitral tissue damage (100, 101) or even SAM (systolic anterior motion) of the anterior mitral leaflet (102).

Skirting a stent that comprises diverse design elements creates greater challenges than covering stents that predominantly consist of repetitive elements. For example, crimping of our scallop-based stent leads to areas where the skirt elongates up to 67%, which considerably exceed the maximum estimated strain of 50% for the skirt of the Sapien 3 Ultra [based on Yudi et al. (103)] and 42% for the Evolut Pro [based on Jubran et al. (104)]. Electrospinning the skirt from the same polymer as used for the leaflets provided both the required viscoelasticity and an ingrowth-permissible porosity. By fully covering each supra-annular arm, the skirt becomes an integral part of the physiologically shaped neo-sinuses. The concern that the skirt-covered commissural area may be potentially occlusive in relation to coronary ostia was recently put into perspective in a study using post-implantation CT after both SE and BE TAVR. Although 51% of cases showed severe overlap of the neo-commissures with either or both coronary ostia due to rotational misalignment, no impediment of coronary blood flow occurred (105). Instead, coronary obstruction seems to occur due to displacement of the native valve leaflets by the TAVR stents or by displaced calcium in patients with a low LCA (106). In our XH tests, two features of the SAT stent have been confirmed to increase the safety-space between the edge of the native leafelets and the LCA: the supra-annular arms cause a “downward-tenting” of the native leaflet at the rim of the annulus, thereby lowering the edge of the native leaflets, and the nonagonal cross-sectional footprint created by the stent arms, by countering the congruency in shape that cylindrical stents cause opposite the sinus wall. With a height of 7 mm above the stent-waist (and therefore the annulus), the SAT skirt lies somewhere between the 8–10 mm in the Evolut R/Pro (103, 107) and the 5.7 mm reported for the Sapien 3 Ultra (if implanted in an 80%:20% aorto:ventricular ratio) (108, 109). This lies well below the coronary height of 10.3 ± 1.6 mm that was identified as risk factor for coronary obstruction (106). Furthermore, the three uncovered upper “spacer arms” potentially increase the inflow space even in effaced sinuses of Valsalva that fall into the high-risk group when diameters are less than 27.8 ± 2.8 mm (106). The inward-tilt of the upper part of the SAT as well as the Acurate skirts, compared to an outward-tilt in the Evolut and a zero-tilt in the Sapien further increases the distance to the coronary ostia. Additionally, the upper spacer arms point downwards with their struts in a V-shape. As the lowest point of the “V” lies below the average position of the LCA ostium, this shape should facilitate potential coronary interventions (29, 30, 110).

### Non-occlusive TA Deployment Without Rapid Ventricular Pacing (RVP)

A major feature of the SAT system is the non-occlusive TA balloon-based delivery device. In view of the narrow focus in HICs on predominantly AS patients, any effort to develop an innovative TA deployment system for AR patients may seem anachronistic. Yet, the transapical access had allowed general surgeons to successfully treat RHD making it the commonest heart operation performed in the beginning of the second half of the twentieth century especially in countries of the southern hemisphere. This highlighted the potential of this route to treat valvular heart disease in the absence of open heart surgery (111) making it one of the backbones of our concept.

In TAVR, TF delivery has continually emerged as the dominant access route in AS patients, epitomized in the PARTNER 3 trial where the lack of TF access was an exclusion criterium (3). Nonetheless, this shift was only possible under socioeconomic circumstances that allowed the regular use of another costly secondary device, as TF access requires a vascular closure device at the conclusion of the procedure in practically all patients (112). However, while in calcific AS this trend increasingly restricted TA access to patients with unsuitable iliofemoral vessel size or significant vessel tortuosity (113, 114) the rational for an antegrade approach is entirely different for AR with its high stroke volumes and often significantly decreased ventricular function. Moreover, given the natural history of AR, with its long asymptomatic period and the relatively short transition into decompensated excentric volume-overloaded failure, RVP is unlikely to be tolerated in a significant proportion of these patients. Although an integral part of conventional TAVR procedures for AS, RVP is known for its detrimental effect on the myocardium (21). Every episode of RVP increases the incidence of new atrial fibrillation, acute kidney injury, in-hospital mortality and 1-year mortality (21). In a 2021 study, the significant decrease of cerebral oxygenation observed during RVP was a predictor for the occurrence of neurological complications such as TIA/stroke and post-intervention delirium (115). The Chinese consensus on TAVR recognizes this danger by stating that “the total pacing time should be <15 s to avoid serious complications caused by prolonged hypoperfusion” (53).

While clinical results suggest that in AS, with its concentric hypertrophy, the overall benefits of TAVR still outweigh the harm caused by RVP, this is unlikely to be the case for many patients with symptomatic AR. Once high-volume remodeling has led to endsystolic dimensions of more than 25 mm/m^2^, together with ejection fractions of <35% (116) these patients are considered very high risk. According to a European Heart Study, up to one-fifth of patients with pure AR are in this category (56) with an annual mortality of 10–20% associated with conservative therapy (56) and a 59% post-operative 10 year mortality (116) if operated. The 14% intraoperative mortality of this sizeable patient group highlights the vulnerability of the thinned-out myocardium in response to the ischaemia associated with open heart surgery, even with modern cardioplegia. At the same time, it is unlikely that TAVR that require RVP will cause less damage to an already weakend myocardium. Given the myocardial injury associated with RVP in the concentric hypertrophy of AS (21) rapid ventricular pacing can be expected to have a particularly detrimental effect on the borderline myocardial capacity of the excentric, volume-overloaded ventricles of late diagnosed AR. While some attempts to insert SE TAVR without RVP were successful in patients with AS (117) the need to stabilize the aortic root during deployment seems to be essential for AR. Although patients in HICs usually present for valve replacement well before AR has led to the hallmark hyperdynamic, jerking motion of the aortic root, TAVR could not be performed without RVP with the same procedural success rate as in AS (117). Although 20% of patients receiving an Acurate Neo for native valve AR were rapid-paced, a second TAVR using a balloon-expandable valve was necessary in every 8th patient (54, 118).

Avoidance of RVP during deployment of a BE TAVR can only be achieved if the balloon does not obstruct bloodflow. Hollow-balloons, developed with multiple longitudinal tube structures have recently been used for predilatation of AS (117). However, a helical balloon not only delivers the very high radial force (almost 1,000 N) needed to deploy a BE TAVR (Figure 11) but also has a significantly larger luminal area at comparable outer diameter, as well as half the pressure gradient when fully deployed (unpublished data). During a series of pre-clinical implants of the SAT PU TAVR, the mean gradients during the deployment were 19.4 ± 9.3 mmHg, with peak gradients of 32.0 ± 6.5 mmHg (119). By incorporating the location and stabilization components into the balloon system rather than the valve stent (120), all functions of a sophisticated deployment system for hyperdynamic AR were combined in one device and all were activated by pressure-controlled inflation. Unlike AS, where the TA access route largely reflected the chronology of development, this SAT system was designed for the hyperdynamic AR that is so prevalent in LMICs. Apart from enabling tactile location even with less sophisticated imaging facilities, it stabilizes a hyperdynamic root during unmitigated ventricular contraction. Both these requirements can be easily realized *via* a direct TA access route compared to a long and multiply bent TF system. With the locator/stabilization trunks on the balloon, their pull-line system allows individual height adjustment if required as well as retraction through invagination, making it impossible for the stent to pinch the trunks and get dislodged after deployment, even in very narrow sinuses. The temporary nature of the trunks during deployment allows building them at a diameter of >4 mm, minimizing the risk of leaflet perforation, while keeping generous inflow spaces between the valve and the sinus wall during deployment.

A further feature of this deployment device is the temporary backflow valve. The combination of a large lumen with a temporary backflow valve allows for a slow controlled implantation of the TAVR prosthesis while maintaining physiological aortic pressures throughout (119).

### TF Access and Pre-dilatation for Aortic Stenosis

The SAT system was primarily designed to address the needs of patients with pure AR, even at a late stage of the disease; however, the polymer leaflets represent a potential quantum leap for all recipients of TAVR including patients with AS currently deemed to young. Nonetheless, a transcatheter valve that can only be delivered trans-apically would not be considered for the vast majority of patients in HICs. A universal polymer TAVR, therefore, needs to be also deliverable trans-femorally. While conventional TF delivery devices have been perfected for highly accurate deployment and even integrated rotational alignment (121) the *sine qua non* for this access route is crimpability of the valve to a diameter that can pass through the iliac vessels without detriment. Although the crimp diameter of SE TAVR has been continually reduced, expandable introducer sheaths have overcome the natural disadvantage of BE TAVR which retain a minimum crimp diameter of 20 to 23 Fr (122, 123). Expandable sheaths not only overcome psychological barriers by having diameters as low as 14 Fr while actually expanding to as much as 24.3 ± 1.7 Fr during passage (124, 125), but also have reduced vascular complications. With <20 Fr for the medium-size TAVR the SAT universal stent allows for similar crimp diameters as the most widely implanted TAVR valve and as such is compatible with TF delivery.

Although the combined need for pre- and post-dilatation in patients with AS is still moderately lower in BE than in SE valves (126), modern TAVR systems have lowered the proportion of patients undergoing an additional balloon procedure to one in four (127). Recognizing the detrimental effect of RVP, partially non-occlusive dilatation balloons were used for pre-dilatation in beating hearts (117). Expanding the helical balloon concept of the SAT delivery device to a TF dilatation balloon further extends the system to potential use in patients with AS. Apart from a robust radial force, the main advantage of a helical balloon is it's 44% larger lumen than that of its longitudinal counterpart (Bard Vascular Brochure 2018).

## Conclusions

Middle income countries are catching up fast with TAVR. However, as contemporary products have been conceptualized for the needs of the affluent regions of the world, their use will remain confined to an aging urban population. With so many relatively young patients of emerging economies suffering from AR, there is a need to expand the often limited capabilities to replace heart valves and the next TAVR era will have to address global needs and not just those of the high income countries.

The approach presented in this paper is based on the belief that global needs are better addressed by BE TAVR. The resulting design provided a scallop-shaped core which allows the direct attachment of leaflets, including polymeric leaflets. Elevating arms, extending exclusively on the basis of plastic deformation, secure the anchorage in non-calcified and regurgitant valves. The use of an electrospun skirt allows trans-mural capillary ingrowth, facilitating accelerated surface enothelialization of the neo-sinuses. The back-flow-protected hollow-balloon system of the delivery device with its retractable balloon locator and stabilization-trunks allows slow deployment in a beating heart. Preclinical experience suggests that the avoidance of expensive secondary procedures has been addressed. The tactile non-occlusive delivery system not only makes RVP unnecessary, but also allows implantation in the absence of sophisticated imaging equipment. The stent design allows a crimp diameter that is even smaller than that of the most widely implanted conventional TAVR, also opening the door to TF delivery.

By combining this universal system with polymer leaflets, a powerful disruptive technology for heart valve disease has been incorporated into a TAVR that addresses global needs.

As such, this SAT system fulfills all prerequisites to expand the scope of TAVR to the many more patients living in low- to middle-income countries, while also bringing hope to the patients of high-income countries that are presently excluded from TAVR.

## Data Availability Statement

The raw data supporting the conclusions of this article will be made available by the authors, without undue reservation.

## Ethics Statement

The animal study was reviewed and approved by the Animal Research Ethics Committee of the Faculty of Health Science at the University of Cape Town.

## Author Contributions

HA and KP introduced and executed the novel idea of plastic deformation in a BE stent. PZ and DW wrote the manuscript. BvB, BdJ, JdV, RC, MC, and RS: development. PZ, DB, DW, and HC involved in the daily R&D and production. PH: statistical oversight and design of animal verification experiments. JSc, CO, JSw, FV, BP, CS, LC, HT, HS, TF, MG, XL, and SM actively provided clinician feed-back with involvement in tests, animal experiments and modifications. All authors contributed to the article and approved the submitted version.

## Conflict of Interest

“Strait Access Technologies” (SAT) is a start-up company of the University of Cape Town. HA, DB, JSc, HC, DW, and PZ own shares in SAT. JV, RC, and RS are employed by SAT. KP, BB, and MC are former employees and shareholders of SAT. BJ is a former employee of SAT. CS is employed by Auto Tissue Berlin. The remaining authors declare that the research was conducted in the absence of any commercial or financial relationships that could be construed as a potential conflict of interest.

## Publisher's Note

All claims expressed in this article are solely those of the authors and do not necessarily represent those of their affiliated organizations, or those of the publisher, the editors and the reviewers. Any product that may be evaluated in this article, or claim that may be made by its manufacturer, is not guaranteed or endorsed by the publisher.
